# Comparative rhizotaxy of fossil and living isoetalean rhizomorphs reveals development through rootlet intercalation within a triangular lattice

**DOI:** 10.1093/aob/mcaf277

**Published:** 2025-10-31

**Authors:** Jeremy Wyman, Richard M Bateman, Liam Dolan, Jennifer L Westermann, Alexander J Hetherington

**Affiliations:** Institute of Molecular Plant Sciences, School of Biological Sciences, University of Edinburgh, Max Born Crescent, Edinburgh EH9 3BF, UK; Jodrell Laboratory, Royal Botanic Gardens, Kew, Richmond TW9 3DS, UK; Gregor Mendel Institute of Molecular Plant Biology (GMI), Austrian Academy of Sciences, Vienna Biocenter (VBC) Vienna 1030, Austria; Institute of Molecular Plant Sciences, School of Biological Sciences, University of Edinburgh, Max Born Crescent, Edinburgh EH9 3BF, UK; Institute of Molecular Plant Sciences, School of Biological Sciences, University of Edinburgh, Max Born Crescent, Edinburgh EH9 3BF, UK; Royal Botanic Garden Edinburgh, Edinburgh EH3 5LR, UK; Department of Natural Sciences, National Museums Scotland, Edinburgh EH1 1JF, UK

**Keywords:** Root, root evolution, rhizotaxy, rhizomorph, Isoetales, *Stigmaria*, *Oxroadia*, *Isoetes*, Carboniferous

## Abstract

**Background and Aims:**

Isoetales is a clade of lycopsids that evolved colossal arborescent forms during their Palaeozoic prime but today are represented solely by the small, herbaceous monogeneric *Isoetes*. Despite the differences in scale of taxa in the clade, the rooting system of all members consists of two parts; rootlets develop from a rhizomorph in a regular pattern termed rhizotaxy. Rhizomorphs are highly diverse in morphology, leading to different terms being used to describe aspects of rhizotaxy in contrasting lineages. Here we set out to investigate the degree to which rhizotaxy was conserved among taxa, aiming to provide a standard geometric definition and developmental interpretation of rhizotaxy.

**Methods:**

We developed a pipeline to quantitatively describe rhizotaxy. This pipeline allowed rootlet arrangement to be captured in 3D, before being visualized on a 2D lattice to which Delaunay triangulation could be applied. This approach offers a standard quantitative method of comparing rhizotaxy across disparate rhizomorphs. Next, to investigate the evolution and development of rhizotaxy we applied our pipeline to 3D reconstructions we generated of the rooting system of the extinct Carboniferous lycopsid, *Oxroadia*. Finally, we made direct observations of rootlet development in *Isoetes* using time-course imaging.

**Key Results:**

We demonstrate that rhizotaxy can be described as an equilateral triangular lattice for all members of the Isoetales, including *Oxroadia*. By combining evidence from direct observation of rootlet development in *Isoetes* with inferences of rootlet development and the early stages of sporophyte ontogeny of *Oxroadia*, we conclude that the conserved rhizotaxy developed through the process of rootlet intercalation.

**Conclusions:**

We provide a single geometric definition and predicted developmental mechanism for rhizotaxy that applies to all Isoetales. Our findings call into question the literal interpretation that the rhizomorph is a modified shoot.

## INTRODUCTION

During the Pennsylvanian subperiod (333–298 Mya), Euramerican terrestrial ecosystems were periodically dominated by extensive peri-equatorial swamps famously occupied by giant arborescent lycopsids such as *Lepidodendron* and *Sigillaria*. These remarkable trees arguably marked both the evolutionary and ecological high-water marks of the lycopsids (DiMichele and Phillips, 1995; Hilton and Cleal, 2007; DiMichele, 2014; Montañez *et al.*, 2016), being highly adapted to a range of wetland niches (Bateman *et al*., 1992; Phillips and DiMichele, 1992). One key feature widely considered to have contributed to their success was the evolution of a unique rooting system termed a rhizomorph. This rooting system is a synapomorphy of the Isoetales, a derived clade of lycopsids that includes extinct taxa from the Devonian onwards, as well as the extant genus *Isoetes*. Rhizomorphic rooting systems consist of two distinct categories of organ: the rhizomorph and the associated rootlets. Rhizomorphs are diverse in form; they can be radially symmetric, woody and several metres in length, as in *Stigmaria*, shallow with a highly branched vasculature but tiny in size, as in *Oxroadia*, or bilaterally symmetric and compacted, forming cormose forms, as in *Pleuromeia* and *Isoetes* (DiMichele and Bateman, 1992, 1996; Pigg, 2001; Thomas and Seyfullah, 2015; DiMichele *et al*., 2022). By contrast, rootlets are always radially symmetric in outline, with a characteristic large internal lacuna, though internally their vascular anatomy and connective linking vasculature to cortex render the rootlets strongly bilateral. They branch dichotomously and develop root hairs. Investigations of *Isoetes* have demonstrated that rootlets are anatomically, developmentally and genetically similar to the roots of other species within the lycopsid clade (Yi and Kato, 2001; Hetherington and Dolan, 2017; Hetherington *et al*., 2020), suggesting they are homologous and conserved across lycopsids. Here, we have chosen to refer to these organs as rootlets rather than roots only for consistency with the published literature, rather than to imply any distinction from the roots of other lycopsids. By contrast, we accept that the rhizomorph is unique to the clade, though highly variable among taxa. Despite this variability, all rhizomorphs incorporate long-lived meristems that give rise to rootlets in a regular pattern termed rhizotaxy.

The concept of rhizotaxy is derived from the concept of phyllotaxy – the arrangement of leaves on aerial axes of plants – but is instead used to describe the pattern of rootlet arrangement. Despite rhizotaxy being a highly conserved feature of the group, the terminology used to describe it has differed between stigmarian and cormose rooting systems. On axial rhizomorphs of *Stigmaria*, emergent rootlets are described as helices which have been characterized by the presence of ‘parastichies’ (diagonal relationships) and ‘orthostichies’ (vertical relationships). By contrast, in cormose forms such as *Isoetes*, rootlets do not form helices and have instead been described as forming ‘series’ and orthostichies. This difference in terminology is frustrating when seeking developmental similarities in the group, so one aim of our study is to adapt geometric approaches, developed for descriptions of phyllotaxy (Eithun *et al*., 2019; Godin *et al*., 2020), in order to generate a simplified geometric definition of rhizotaxy that can be applied to all rhizomorphic rooting systems.

Using this approach, we then apply our new technique to one of the most distinct rhizomorphic rooting systems recorded – the rooting system of the early Carboniferous *Oxroadia* (Bateman, 1992, 1994). This genus occupies an important phylogenetic position as an early-diverging member of Isoetales, as well as spanning a transitional period during the rise to ecological dominance of the wetland environments synonymous with the Middle–Late Carboniferous (Millward *et al*., 2018; Scott, 2024). *Oxroadia* was determined to be a member of the isoetalean clade through a combination of characters; it was heterosporous, produced secondary xylem in the stem, and developed a two-part rooting system with distinct rhizomorph and rootlets. Although its phyllotaxy could be determined (Bateman, 1992), its minuscule and highly branched rhizomorph precluded accurate identification of its rhizotaxy. Therefore, *Oxroadia* remains an ideal test case to investigate if rhizotaxy is a ubiquitous feature of the group or, based on its phylogenetic position, if it is possible that rhizotaxy was a later innovation within the isoetalean lineage.

Our final reason for re-investigating *Oxroadia*’s rooting system is its value for addressing, arguably, the most significant remaining question about Isoetales: the evolutionary origin of the rhizomorph. A long-standing hypothesis suggests that the rhizomorph represents a remarkable evolutionary transition of a shoot system that has in various ways been modified to carry out the rooting function, resulting from an initial early dichotomy of the embryonic axis (underlying issues discussed by Phillips, 1979; Stubblefield and Rothwell, 1981; Rothwell and Erwin, 1985; Phillips and DiMichele, 1992; Bateman, 1994; DiMichele *et al*., 2022). The evidence supporting this hypothesis is based primarily on the discovery of the exceptionally well-preserved embryo of the Carboniferous tree-clubmoss *Lepidophloios* interpreted to have preserved this initial dichotomy (Stubblefield and Rothwell, 1981; Rothwell and Erwin, 1985; Rothwell *et al*., 2014; D’Antonio and Boyce, 2020; DiMichele *et al*., 2022). By contrast, the embryos of extant *Isoetes* provide no evidence of this initial dichotomy (Paolillo, 1963; Yi and Kato, 2001; Jiang *et al*., 2015) and instead more closely resemble the embryos of other lycopsids (Bower, 1935; Foster and Gifford, 1974; Pigg, 2001; Tomescu, 2010). As both of these taxa are highly derived within the Isoetales, and they develop distinct rhizomorphs, it is difficult to judge which of these genera provides greater insight into the evolutionary origin of the rhizomorph. *Oxroadia* provides a unique perspective into this debate because of its putatively, relatively primitive phylogenetic position among the rhizomorphic taxa (Bateman *et al*., 1992; Bateman, 1994), and because both juvenile and embryonic fossils have been described (Long, 1971; Bateman, 1992). By re-investigating this important member of Isoetales, we therefore aim to develop new methods for quantifying rhizotaxy, establish if a definable rhizotaxy was present, and predict the subterranean ontogeny of *Oxroadia*.

## MATERIALS AND METHODS

### Fossil material

This study is based on the re-investigation of three published *Oxroadia* rooting systems: *Oxroadia* sp. (Long, 1971), *O. gracilis* (Long, 1986) and *O. conferta* (Bateman, 1992). During the primary description of each specimen, both of the original authors first cut specimens into slabs, and then numerous serial peels were prepared from the cut surfaces using the acetate peel method (Joy et al., 1956). Our investigation focused on a subset of the peels for each specimen. *Oxroadia* sp. (Long, 1971) slides AGL7340–7415d Great North Museum, *O. gracilis* (Long, 1986) slides NMS G.1984.71.(1-74) National Museums Scotland and *O. conferta* (Bateman, 1992) peels OBC01c2T–OBC60c1B University of Birmingham.

Alongside our re-investigation of *Oxroadia*, we examined four specimens of the larger-bodied *Stigmaria ficoides* in the collections of National Museums Scotland, accession numbers NMS G.2025.4.1, G.1988.15.1, G.2022.11.49 and G.1983.34.2.

### Geological setting

All three *Oxroadia* specimens were calcareous permineralizations from the late Tournaisian (Mississippian: ∼350 Mya) of SE Scotland. *Oxroadia* sp. (Long, 1971) was discovered alongside the Whiteadder River at Blue Scaur (Grid Ref. NT815565), and the two other specimens (Long, 1986; Bateman, 1992) were both discovered at Oxroad Bay (Grid Ref. NT598848), the locality from which the type specimens of *Oxroadia* (Alvin, 1965; Bateman, 1992) were discovered. Three specimens of *Stigmaria ficoides* (NMS G.2025.4.1, G.1988.15.1 and G.1983.34.2) were also found in southern Scotland, but they date from the Visean stage, around 10 million years younger. The fourth *Stigmaria* specimen (NMS G.2022.11.49) lacks associated metadata.

### Living material

We investigated living material from two species of *Isoetes*, *I. lacustris* and *I. echinospora*, as well as reinvestigating the previously figured *I. howellii* (Paolillo, 1982). *Isoetes echinospora* plants were grown submerged in aquaria in Levington M2 compost topped with coarse gravel in a glasshouse at Oxford University. *Isoetes lacustris* was cultivated at the Institute of Molecular Plant Sciences at the University of Edinburgh. It was grown in a half sand, half peat matrix within a tank full of tap water. For time lapse images, mature plants were removed from the soil, washed and grown in tap water in plastic pots. Rootlet initiation was imaged by inverting mature specimens under a Leica M165 FC stereomicroscope with illumination provided by a ring light. Both species were also investigated through scanning electron microscopy (SEM). *Isoetes lacustris* was fixed using 4F:1G (McDowell and Trump, 1976), and dehydrated to prepare for gold coating and imaging using a Zeiss Crossbeam 550 FIB-SEM. *Isoetes echinospora* was fixed in dry methanol, critical-point dried using a Tousimis Autosamdri-815, treated with a gold/palladium mixture using a Quorum Technologies SC7640 sputter coater, and imaged with a JEOL JSM-5510 SEM. The investigation of *I. howellii* was based on a previously published detailed line drawing showing rootlet arrangement on the base of the corm of *I. howellii* (Fig. 1A of Paolillo, 1982).

### Generating 3D models of *Stigmaria* and *Isoetes* using photogrammetry

To digitally measure rhizotaxy, we first used photogrammetry to capture the 3D structure of four specimens of *Stigmaria* that have a known rhizotaxy. The *Stigmaria* specimens were placed in a Neewer light box and rotated using a manual turntable. Specimens were photographed, in total, 100 times from five different heights at varying angles using a Canon EOS 5D Mark IV camera with a 100-mm macro lens. Images were then imported into Agisoft Metashape Professional 1.8.3 to generate a 3D model. In this software, the images were aligned, then a dense point cloud was generated, and a mesh was built using the ‘highest quality’ setting to ensure the most accurate 3D model (the resulting 3D models were uploaded to Edinburgh Datashare DOI: 10.7488/ds/7972). The model was then exported as both .OBJ and .STL files.

We also investigated rhizotaxy on a 3D model of the corm of *I. lacustris.* The corm was too small for conventional photogrammetry, so we used a method derived from Ball *et al*. (2017) to make a 3D model from SEM images. A Zeiss Crossbeam 550 FIB-SEM was used to take 32 images of the specimen. Images were taken from ten different angles and three rotations per angle for a total of 30 images. The same pipeline used for *Stigmaria* photogrammetry was used to generate a 3D model from these images. (The resulting 3D model was uploaded to Edinburgh Datashare DOI: 10.7488/ds/7972.)

### Generating 3D reconstructions of *Oxroadia* specimens

To generate 3D reconstructions of rooting systems we imaged peels of *O. gracilis* and *O.* sp. using an Epson Perfection Photo scanner at 3200 dpi and photographed the *O. conferta* peels using a Nikon SMZ18 stereo microscope (images of all peels are uploaded to Edinburgh Datashare DOI: 10.7488/ds/7972). We next used the images of the peels to generate 3D reconstructions using the Serial Paleontological Image Editing and Rendering System (SPIERS) software suite (Sutton *et al*., 2012). Peels were manually aligned using SPIERSalign. We next manually masked the external surface and internal vascular tissue of each specimen in SPIERSedit using a combination of the curve and brush tool.

During the masking process we distinguished between stem, rhizomorph and rootlets by their distinct xylem structure. The stem is protostelic whereas the rhizomorph has a siphonostele with a distinctive pith and a xylem. Rootlets have a fan-shaped bundle of monarch protoxylem composed of radial files of metaxylem. When generating 3D models, the thickness of peels was calculated to be 0.2, 0.2 and 0.3 mm for the *O. gracilis*, *O. conferta* and *O.* sp. specimens respectively. The thickness of 0.2 mm for *O. gracilis* and *O. conferta* was based on Long (1986) and Bateman (1988). To estimate peel thickness for these specimens we divided the thickness of the original block by the number of peels that were made from it. The pixel/mm ratio of the peels was measured in ImageJ. To account for the shrinkage of peels during the preparation process, we corrected all our measurements by a factor of 1.05 (Long *et al*., 2022). 3D models were generated using SPIERSview and each element was exported as an .STL file (all 3D models are uploaded to Edinburgh Datashare DOI: 10.7488/ds/7972).

### Describing rhizotaxy using a new digital method

Our models provided a unique opportunity to investigate rhizotaxy in 3D. Our method for this was based on the method established by Turner *et al.* (2023) to quantify phyllotaxy in *Asteroxylon mackiei*, a primitive lycopsid from the Devonian Rhynie Chert. The original method consisted of placing a digital 3D cylinder around the shoot axis and then marking the position of leaves. Next, this cylinder was digitally unwrapped, providing a planar representation of leaf arrangement. We predicted that we could use an updated version of this approach to successfully interpret rootlets on the rhizomorph.

The phyllotaxy of *O. gracilis* had previously been determined directly by means of fully exposing the cylindrical surface of a leafy branch to obtain a complete tangential acetate peel (text-fig. 5 of Bateman, 1992). Unlike Turner *et al*.’s (2023) study quantifying leaves on an unbranched stem, the highly branched nature of the rhizomorph of *O. gracilis* and *O. conferta* meant it would not be possible to apply a single global cylinder around the rhizomorph to investigate rhizotaxy. We therefore first subdivided our 3D reconstructions of *O. gracilis* and *O. conferta* into individual segments of the rhizomorph for further investigation. This allowed us to quantify rhizotaxy on a short unbranched segment of the rhizomorph. Next, as most tissues other than the xylem were damaged by decay and calcite/dolomite recrystallization, we performed our analysis solely on the xylem trace rather than the emergence of rootlets from the rhizomorph surface.

All four *Stigmaria* models, the *Isoetes* model and the seven *Oxroadia* rhizomorph segments were imported into the 3D modelling software Blender for imaging and measuring. Because the *Oxroadia* peels preserve the exact point that the rootlet vascular bundle emerges from the rhizomorph vascular stele, the mesh cylinders were placed around each individual *Oxroadia* rhizomorph branch reconstruction and scaled until they fit tightly around the vascular trace of the rhizomorph. The circumference and height of the cylinder were measured using the ‘ruler’ tool in Blender. A segment of the cylinder mesh that did not intersect vertically with a rootlet was chosen to be the seam from which the 3D cylinder was subsequently ‘unwrapped’.

**Fig. 1.**
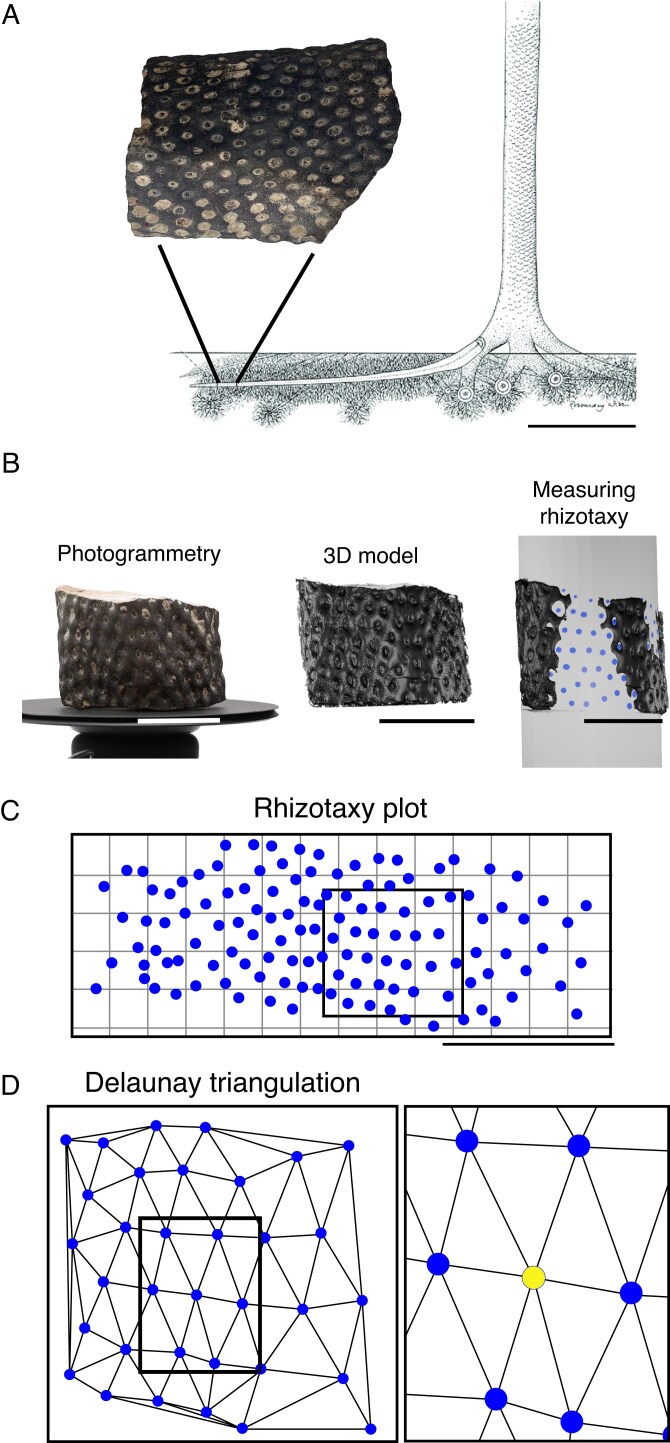
Quantification of stigmarian rhizotaxy. (A) The rhizomorph of the arborescent lycopsids consists of large woody axes and is named *Stigmaria* (source: Hetherington *et al*., 2016). (B) The 3D structure of *Stigmaria* was captured with photogrammetry and the positions of rootlet scars were marked on a digital cylinder. (C) The cylinder was then digitally unwrapped to display rhizotaxy on a 2D lattice. (D) Delaunay triangulation was applied to a small region of the rhizotaxy plot, revealing a characteristic equilateral triangular lattice. Each rootlet, such as the one highlighted in yellow, has connections to those on horizontal series above and below it. Scale bars: (B), (C) = 6 cm.

**Fig. 2.**
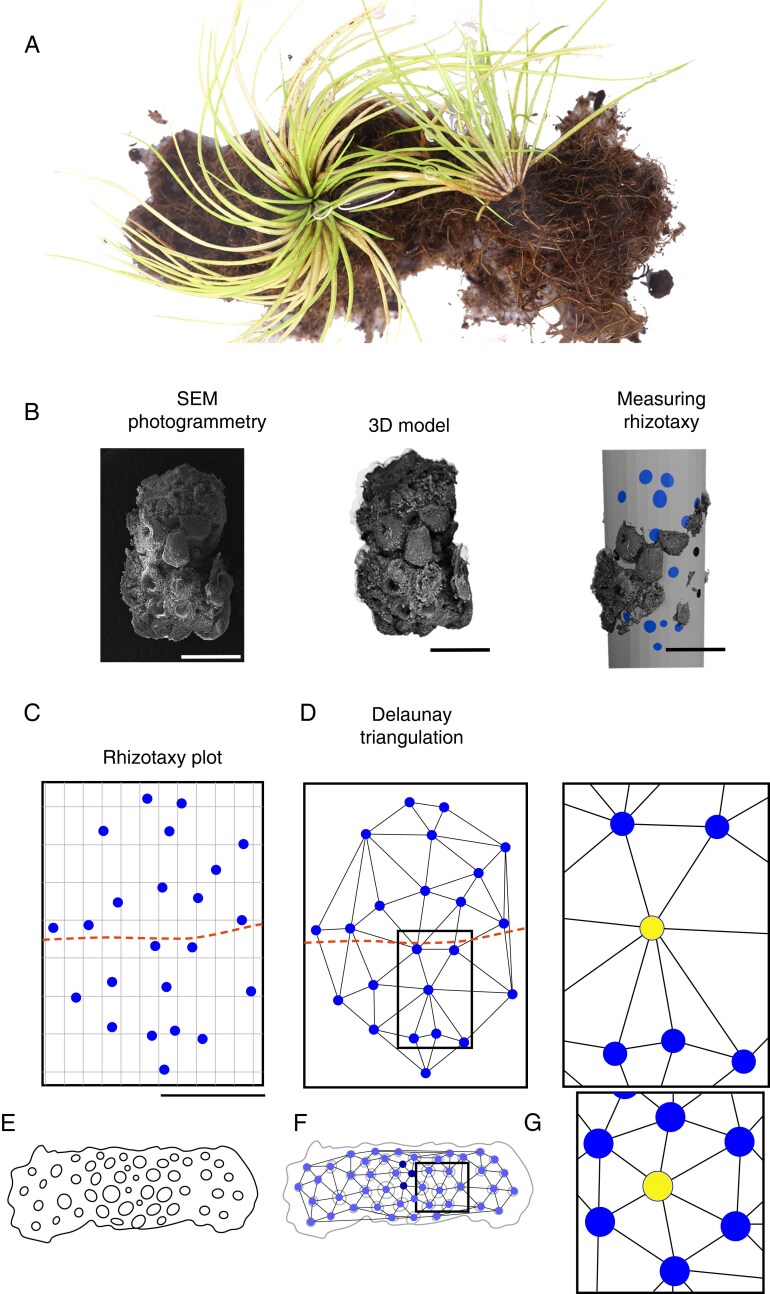
Quantification of *Isoetes* rhizotaxy. (A) The rhizomorph of *I. echinospora* subtending the leaves is small and cormose. (B) The 3D structure of the rhizomorph was captured using SEM photogrammetry, allowing us to mark the points of rootlet attachment in 3D. (C) The position of the rootlets was displayed on a 2D lattice. (D) Delaunay triangulation was applied to the rhizotaxy plot, demonstrating the rhizotaxy on an equilateral triangular lattice. The enlarged region of the Delaunay triangulation shows the relationship between the rootlet in yellow and rootlets occurring on successive horizontal series above and below. The red dashed line (C, D) marks the position of the rhizomorph meristem and central furrow. (E–G) Investigation of rhizotaxy on the published image of *I. howellii* (fig. 1A of Paolillo, 1982). Scale bars: (B), (C) = 3 mm.

A white base colour texture was added to the cylinder and modified using the ‘Texture Paint’ mode in Blender. The models remained static while we rotated the viewport in 3D space to mark the points at which rootlets or rootlet scars intersected with the cylinder; points were recorded as a blue or black dot using the brush tool (see Figs 1B and 2B). To visualize the position of these dots on a planar 2D representation of the 3D cylinder, we switched to the ‘UV Layout’ mode in Blender. Each brush mark on the cylinder was represented by a corresponding dot on the white texture displayed in the UV layout. The resulting image displays the pattern of rootlet arrangement and is referred to as a rhizotaxy plot. These were exported from Blender as PNG image files. Rhizotaxy plots were imported into RStudio using the ‘imager’ package and simplified into black and white images using a threshold that determined the intensity of each pixel, thereby rendering darker pixels such as blue dots black and lighter pixels white. Next, we used the ‘imager’ package to turn the rhizotaxy plots from images into quantitative graphs where relationships between rootlets could be quantitively characterized (see Figs 1C and 2C).

### Describing rhizotaxy in Isoetales using Delaunay triangulation

Delaunay triangulation has been shown to accurately represent the contact parastichies between primordia when investigating phyllotaxy using a cylindrical approach (Eithun *et al*., 2019). This approach can be adopted for measuring rhizotaxy, which is traditionally measured using parastichies and orthostichies. Using the ‘deldir’ package, Delaunay triangulation was applied to the coordinates of the previously generated, scaled centroids. Because hundreds of rootlets are emitted by each small section of *Stigmaria*, applying Delaunay triangulation to the whole rhizotaxy plot would have proven uninformative, so a random part of each rhizotaxy plot was chosen. Irregular white spaces in the middle of the rhizotaxy plot reflect fossil deformation (as evident in Supplementary Data Fig. S1A). Because *Isoetes* and *Oxroadia* produced fewer rootlets, Delaunay triangulation could be applied to the whole rhizotaxy plot.

Although Delaunay triangulation is a useful tool for visualizing a regular rhizotaxy, the geometric process precludes points from being located inside the circumcircle of any triangle, meaning that any local variation can cause the formation of either hexagonal or pentagonal patterns, depending on a ‘central point’. In reality, this distinction does not reflect a change in the underlying pattern but rather is an artefact of the triangulation process. Delaunay triangulation maximizes the smallest angle of the triangle but does not guarantee an equilateral triangle so, using the ‘deldir’ and ‘geometry’ packages, the angles of the vertices of each triangle were measured and averaged. An equilateral triangular lattice rhizotaxy can thereby be interpreted from the triangular components of the hexagonal and pentagonal patterns of parastichies.

## RESULTS

### Digital quantification enables successful identification of equilateral triangular lattice rhizotaxy in *Isoetes* and *Stigmaria*

To establish whether digital methods for quantifying rhizotaxy could capture the same results as classical approaches (Paolillo, 1963; Karrfalt and Eggert, 1978; Charlton and Watson, 1981), we first investigated *Stigmaria* and *Isoetes* – members of the Isoetales with known rhizotaxy. The 3D structures of four *Stigmaria* specimens were modelled using photogrammetry, and rhizotaxy was quantified using our new pipeline (Fig. 1A–D; Supplementary Data Fig. S1). This approach allowed us to investigate rhizotaxy rapidly across a range of *Stigmaria* specimens. We noted some variation in the overall distribution and density of rootlet scars among specimens (Fig. 1C; Fig. S1), but the majority of this variation reflected compression or damage of the originally cylindrical axis producing a higher density of rootlet scars towards the centre and edge of the specimen (Fig. 1C; Fig. S1). For our in-depth analysis of rhizotaxy we focused on one smaller area that was not damaged or distorted due to preservation and again applied Delaunay triangulation (Fig. 1D). Delaunay triangulation helped visualize the rhizotaxy in an unbiased way and allowed us to draw conclusions about the position of each rootlet relative to those that surround it. When an individual rootlet is taken as a starting point, such as the rootlet highlighted in yellow in Fig. 1D, evident to the left and right of this rootlet will be two rootlets on an approximately horizontal or very low pitch line. This line has been referred to by previous authors as a paragenetic spiral (Charlton and Watson, 1981). The yellow rootlet also has connections to four further rootlets, two above and two below, connected by diagonal lines. These diagonal lines have been referred to by previous authors as parastichies. This pattern of rootlet emission is highly conserved across *Stigmaria* and has also been described using classical approaches (Charlton and Watson, 1981). Our approach demonstrates the utility of using Delaunay triangulation to identify the paragenetic spiral and parastichies in an unbiased way. The only feature used in classical descriptions of rhizotaxy that is not captured through Delaunay triangulation are lines termed orthostichies that are perpendicular to the paragenetic spiral. However, as they are perpendicular to the paragenetic spiral, they provide no additional information toward the quantification of local rhizotaxy (they can also be added manually to the rhizotaxy plots if subsequently needed).

There are several ways that the rhizotaxy of *Stigmaria* has previously been described, such as forming helical arrangements of rootlets described by quantifying features of the parastichies. However, based on our investigation using Delaunay triangulation, a more straightforward and versatile approach is to describe this pattern as an equilateral triangular lattice. This lattice is composed of multiple roughly parallel lines of rootlets, with the position of rootlets on successive lines offset so that a rootlet (such as the one highlighted in yellow in Fig. 1D) will sit between two rootlets above and two below. Finally, we can quantify this lattice by calculating the angles formed between the vertices of the triangles in the Delaunay triangulation. Applying this procedure to all the rhizotaxy plots for *Stigmaria* indicates that the most frequent angle found in this analysis is roughly 60°, indicating that the majority of triangles are equilateral (Supplementary Data Fig. S2). Our new approach to quantifying rhizotaxy indicates that stigmarian rootlets are arranged as an equilateral triangular lattice rhizotaxy.

**Fig. 3.**
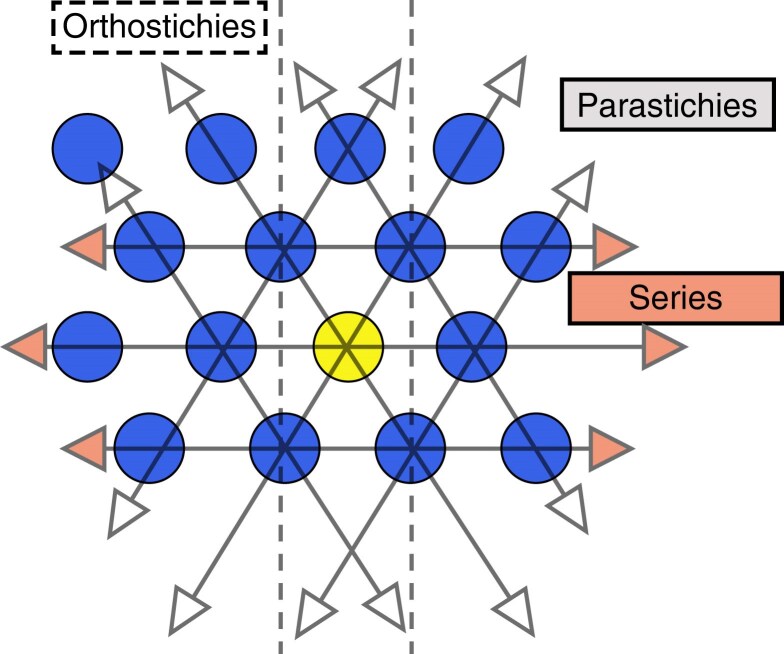
Delaunay triangulation allows the visualization of both parastichies and series as a new way to evaluate rhizotaxy across all isoetaleans. Rootlets are represented by circles, with the yellow rootlet used as a central reference point relative to all other rootlets in blue.

We next used a similar approach to quantify rhizotaxy in two extant species, *I. lacustris* and *I. howelii* (Fig. 2). We produced a 3D model and then generated a rhizotaxy plot for *I. lacustris* (Fig. 2A–D) and additionally reinvestigated through Delaunay triangulation the rhizotaxy on the published specimen of *I. howelii* (Paolillo, 1982) (Fig. 2E–G). Our approach again provided an unbiased method to investigate rootlet patterning in *Isoetes*, and we were able to recognize the regular rhizotaxy previously described (Paolillo, 1963, 1982). Similar to *Stigmaria*, the pattern can be described as a triangular lattice composed of horizontal and diagonal lines. In both *Isoetes* specimens, a rootlet, such as the one highlighted in yellow (Fig. 1D, G), will sit adjacent to others on a roughly horizontal line (referred to by previous authors as a series: Paolillo, 1963), and will be connected via diagonal lines to two above and two below. Despite the vastly contrasting scales of the rooting systems of *Stigmaria* and *Isoetes* and the differences in rhizomorph meristem structure that gives rise to rootlets, the overall rhizotaxy at a local scale is conserved as a triangular lattice (Fig. 3). This rhizotaxy includes roughly horizontal lines, the paragenetic spiral of *Stigmaria* (Charlton and Watson, 1981) or series of *Isoetes* (Paolillo, 1963), and diagonal lines termed parastichies (Fig. 3). Having established the success of using our pipeline to describe rhizotaxy in *Stigmaria*, we next applied the technique to the unknown rhizotaxy of *Oxroadia*.

### Identifying portions of *Oxroadia*’s rhizomorph for quantification

**Fig. 4.**
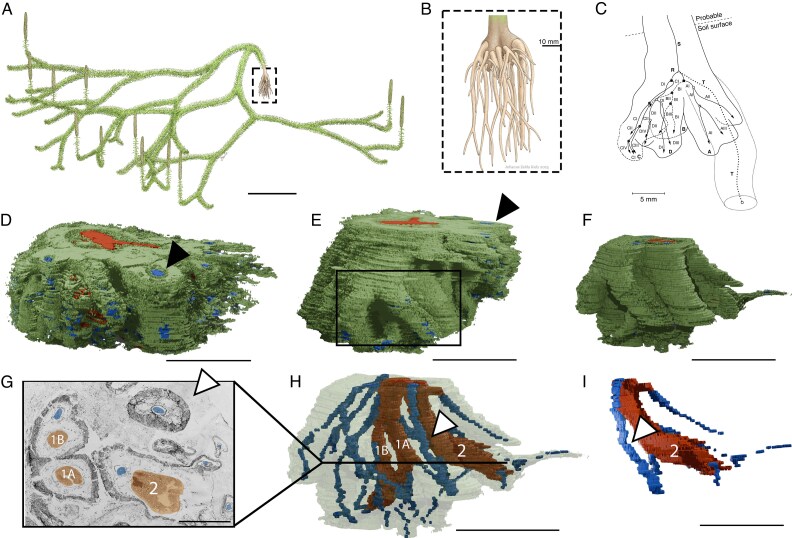
3D reconstructions elucidating the complex two-part rooting system of *Oxroadia.* (A, B) Whole-plant reconstruction of *Oxroadia* with magnified inset of the rooting system (B) (artwork from Julianne Kiely). (C) Reconstruction showing the branching rhizomorph of *Oxroadia conferta* (text-fig. 4A of Bateman, 1992). (D–I) 3D reconstructions of *O. gracilis* generated from serial peels, where green represents the outer cortex of the rooting system, red the rhizomorph vasculature and dark blue the rootlet vasculature. Black arrowhead highlights the large tap root. (E) Rotated view of D showing the region of interest magnified in F–I. (F) The region of interest representing rhizomorph branches OG1, 2, 1A and 1B. (G) A peel from the region of interest with the rhizomorph and rootlets highlighted in their respective colours. (H) A transparent model of F showing the underlying vasculature. (I) The vasculature of rhizomorph branch OG2 with all of its connected rootlets. White arrowheads (G–I) highlight the same rootlet in all three images. Scale bars: (A) = 20 cm; (B) = 10 mm; (C) = 5 mm; (D–F, H) = 6 mm; (G, I) = 3 mm.

We created a 3D model of the complete *O. gracilis* specimen based on serial peels (Fig. 4). This scaled model demonstrated the highly compact nature of the *O. gracilis* rooting system, and the overall similarities to the reconstruction of the *O. conferta* rooting system (Fig. 4C reproduced from text-fig. 4A of Bateman, 1992) demonstrate the very small size of *Oxroadia*’s rooting system compared to previous descriptions of the above-ground system (Fig. 4A–C). In the model we were able to distinguish the overall external surface and internal vascular system; we could also recognize a single prominent rootlet previously referred to as the ‘tap root’ (black arrowhead in Fig. 4A, D, E). Next, using our 3D model, we were able to search for specific areas of the rhizomorph where rhizotaxy could most effectively be quantified. The highly branched nature of the rhizomorph obscured the rhizotaxy so we searched for largely unbranched portions of the rhizomorph where multiple rootlets could be investigated along a single rhizomorph segment. An example of one of these areas is highlighted in Fig. 4E, encompassing what were originally numbered rhizomorph branches 15 and 16 in the description by Long (1986). We refer to these branches here as OG1 (15) and OG2 (16). OG1 dichotomizes to form daughter branches OG1A and OG1B. A high-resolution model of this region of the rooting system was reconstructed and fully segmented in SPIERSedit (Sutton *et al*., 2012) to ensure accuracy when measuring rhizotaxy and distinguishing between rhizomorph and rootlets (Fig. 4F–I). This careful anatomical investigation was needed as the rhizomorph vascular steles dichotomized multiple times before full separation of the branches (Fig. 4G). This behaviour, combined with similarities in sizes between the branches of the rhizomorph and the rootlets, made distinguishing among them based on external characteristics challenging. To ensure we could confidently distinguish branches of the rhizomorph from rootlets and accurately map the position of rootlet emergence, we focused our investigation on branching of the vascular traces. Rootlets and rhizomorphs developed distinct vascular architecture, so they could be readily distinguished in peels, allowing us to accurately map branching based solely on vascular anatomy (Fig. 4G). OG1 dichotomized into rhizomorph branches, with one rootlet attached to branch 1, three attached to OG1B, four attached to OG1A and seven attached to OG2 (Fig. 4H). We then digitally separated individual segments of the rhizomorph (Fig. 4I) which allowed us to quantify rhizotaxy in unbranched portions of the rhizomorph and restricted this analysis to portions of the rhizomorph bearing at least four rootlets. We carried out a similar investigation on a second *Oxroadia* species, *O. conferta*, but only reconstructed one additional rhizomorph segment due to the even higher density of branching in this more compact species. In total, this approach provided six segments of *Oxroadia*’s rhizomorph to quantify rhizotaxy: five branches of the rhizomorph of the *O. gracilis* specimen (OG1A, 1B, 2, 3, 4) and one branch from the *O. conferta* specimen (OC1). We note that the application of this approach would be best suited for specimens with a higher number of rootlets, something not possible to investigate with the highly branching rhizomorphs of *Oxroadia*.

### Quantitative assessment indicates that *Oxroadia* developed a rhizotaxy

**Fig. 5.**
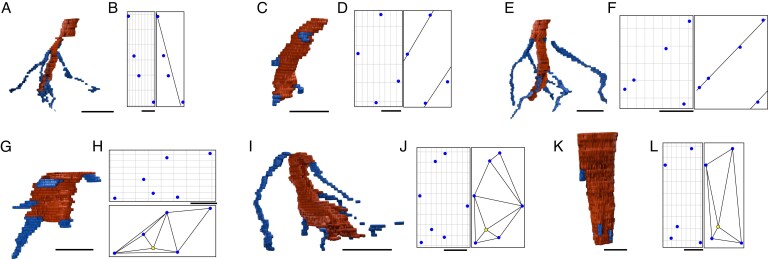
*Oxroadia* rootlets developed in isoetalean rhizotaxy. (A–L) 3D reconstructions of rhizomorph and rootlet vasculature used for quantitative assessment of rhizotaxy; red denotes rhizomorph vasculature and dark blue denotes rootlet vasculature. (A, C, E) 3D reconstructions of rhizomorph branches OG1B, OG1A and OG4B, all of which have four or fewer rootlets on the individual branches. (B, D, F) Rhizotaxy plots demonstrating the arrangement of rootlets in simple helices. The first rootlet on the rhizotaxy plots (A, C) is the same rootlet that emerges immediately at the point where the rhizomorph dichotomizes. (G, I, K) 3D reconstructions of OG3, OG2 and OC1, all of which have five or more rootlets. (H, J, L) The respective rhizotaxy plots, with Delaunay triangulation applied, demonstrate a rhizotaxy composed of parastichies, orthostichies and series. Scale bars: (A, B, I) = 4 mm; (C, D, G) = 2 mm; (E, F, J, L) = 3 mm; (H) = 2.5 mm; (K) = 1.5 mm.

Qualitative assessment of the rhizotaxy on each segment revealed considerable variation among specimens (Fig. 5), including the length of segments, the number of rootlets on each segment and the size of the rhizomorph stele compared with the rootlet steles. There was no consistent number of rootlets per millimetre length of the rhizomorph. The number of rootlets was neither dependent on branch length nor on whether it was a terminal portion of the rhizomorph. For example, the rhizomorph branch OG3 of *O. gracilis* (a dichotomizing branch 3.74 mm in length: Fig. 4G) emits six rootlets whereas OG1B (a terminal branch 7.29 mm in length) emits only four rootlets (Fig. 4A; Table 1). Similarly, the diameter of the rhizomorph is not proportional to the number of rootlets per millimetre. Having established no association between rootlet number and rhizomorph width and length, we next looked for broad qualitative patterns of rhizotaxy. We ruled out the possibility that rootlets occurred in vertical columns or in horizontal whorls, separated by large internodes, based on qualitative investigation of the rhizotaxy plots (Fig. 5). We next investigated whether the rootlets had a preferential orientation of attachment to the rhizomorph. Given the highly branched rhizomorph structure we predicted that rootlets may form preferentially on the outer surface of the rhizomorph facing the substrate, rather than on the internal side in close contact with other rhizomorph branches. We tested this hypothesis in all six rhizomorph segments (Fig. 5) but found that rootlets developed around the full circumference of the rhizomorph, therefore showing no preferential orientation. Despite the variability evident between rhizomorphs, demonstrating that rootlets developed on all sides suggests that rootlets may have emerged in a specific rhizotaxy similar to that of *Stigmaria*.

**Table 1.** Characteristics of the measured rhizomorph branches. A total of 36 rootlets emerged from the seven measured branches of *Oxroadia* rhizomorphs. One rootlet was counted twice for rhizomorph branches OG1A and 1B, because it emerged immediately above the point where the two rhizomorph branches dichotomized from rhizomorph branch OG1.

	*Oxroadia gracilis* 1A	*Oxroadia gracilis* 1B	*Oxroadia gracilis* 2	*Oxroadia gracilis* 3	*Oxroadia gracilis* 4B	*Oxroadia conferta* 1
Terminal	Y	Y	N	N	Y	Y
Number of rootlets	5	4	7	6	4	5
Length (mm)	9.22	7.29	7.80	3.74	5.4	7.13
Diameter of entire branch (mm)	2.8	2.8	4.4	8	3.7	8.8

To test if a triangular lattice rhizotaxy was present in *Oxroadia*, we quantified its rhizotaxy using the same new digital approach (see Figs 1 and 2), producing scaled rhizotaxy plots for each 3D rootlet segment (Fig. 5). We then separated our analysis into segments with fewer than four rootlets and those with more. Two segments possessed only four rootlets (Fig. 5A–D), precluding application of Delaunay triangulation. However, qualitative investigation of both segments suggests that rootlets were arranged as a single helix around the full circumference of the rhizomorph. When viewed on the rhizotaxy plot, a clear diagonal parastichy could be recognized (Fig. 5B, D). We next carried out analyses on the four other specimens that included between five and seven rootlets per segment (Fig. 5E–L). For each of these examples, we applied Delaunay triangulation to the rhizotaxy plots. In all cases, the triangulation helped us to recognize diagonal parastichies in the plots. In OG1A (Fig. 5E, F), the dominant rhizotaxy is also a helical pattern, so we only marked this on the rhizotaxy plot (Fig. 5F). However, for the remaining specimens (Fig. 5G–L) the arrangement cannot be clearly delimited as a single helix. Instead, Delaunay triangulation helped recognize elements of the triangular patterning found in *Isoetes* and *Stigmaria*. Although the small number of rootlets did not allow us to demonstrate the presence of a triangular lattice rhizotaxy, we identified in each specimen evidence of central rootlets, highlighted in yellow (Fig. 5H, J, L), with clear connections to four surrounding rootlets. For each example, the rootlet sits on an intermediate orthostichy between those above and below. This arrangement is significant, as it is a key component of the triangular lattice rhizotaxy described in *Isoetes* and *Stigmaria*. Despite the high variability in *Oxroadia*, both in the number of rootlets per segment and their arrangement, our analysis suggests that rootlets are best considered to develop in a regular rhizotaxy than randomly on the rhizomorph.

Based on our investigation, we conclude that rootlets are arranged in a regular rhizotaxy. This rhizotaxy is more variable and expressed at a much lower density than is found in either *Isoetes* or *Stigmaria* but nonetheless exhibits similarities to both. In all specimens examined, it is possible to identify diagonal lines termed parastichies. In some specimens, especially those with low numbers of rootlets (Fig. 5A–F), this is the only clear component of the rhizotaxy. However, specimens possessing more rootlets (OG2, OG3 and OC1: Fig. 5G–L) show a combination of broadly horizontal and diagonal lines, suggesting evidence of series, parastichies and orthostichies. This result conforms to the overall rhizotaxy described in *Isoetes* and *Stigmaria*.

### Ontogeny in rhizomorphic lycopsid rooting systems

**Fig. 6.**
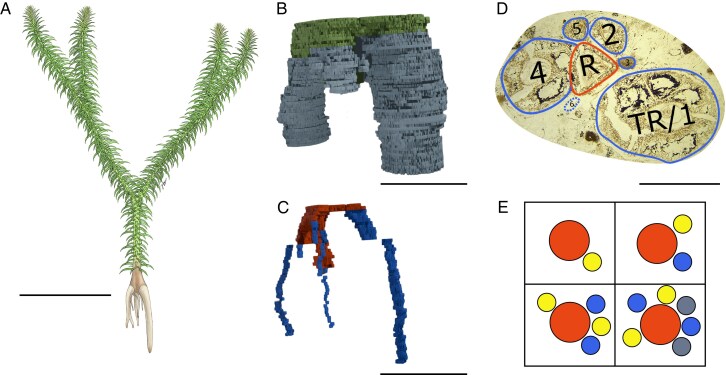
3D reconstruction of the juvenile specimen of *Oxroadia* sp. (derived from Long, 1971) allowing interpretation of rootlet early stages of sporophyte ontogeny. (A) Artist’s reconstruction of the juvenile specimen (artwork by Julianne Kiely). (B–C) 3D reconstruction of the surface and vascular traces (C) of the rhizomorph and rootlets. Green represents the outer cortex of the rhizomorph, light blue the outer cortex of the rootlets, red the rhizomorph vasculature and dark blue the rootlet vasculature. (D) Acetate peel showing five of the rootlets surrounding the central rhizomorph. The rhizomorph is highlighted in red and rootlets in blue; the sixth rootlet that emerges distal to this peel is represented by a small dashed circle where it occurs in relation to the rootlets that are present. (E) Interpretative diagram showing the pattern of rootlet emergence. Rootlets 3 and 4, and 5 and 6, appear to initiate in pairs and bisect the positions of the two preceding rootlets. Scale bars: (A) = 5 cm; (B, C) = 7.5 mm; (D) = 4 mm.

Establishing that *Oxroadia* develops a rhizotaxy opens up the possibility of investigating a fundamental question regarding the ontogeny of the rhizomorph and its rhizotaxy in extinct isoetaleans. There is no consensus because of the paucity of fossils that preserve the early stages of sporophyte development in the juvenile specimens that are needed to connect the earliest stages of sporophyte development with mature forms. To investigate this key transition, we generated a 3D reconstruction and investigated rhizotaxy in, to our knowledge, one of the only juvenile specimens of a Palaeozoic isoetalean (Fig. 6A). We produced a 3D model of the specimen described as *Oxroadia* sp. by Long (1971) (Fig. 6). This 17-cm-long specimen is important because it is regarded as a, more or less, intact juvenile specimen preserving all parts of the plant between the base of the rhizomorph and the shoot tip. Only six rootlets are connected to the unbranched rhizomorph, which remains attached to the stem and the first two orders of dichotomous branching of the leafy shoot. The unbranched nature of the rhizomorph and small number of rootlets mean that by describing their arrangement we can infer the order rootlets developed in the early stages of ontogeny. The 3D reconstruction covered a length of 5 mm (Fig. 6B) and in this zone six rootlets diverged from the rhizomorph vascular trace (Fig. 6C).

Using our 3D reconstruction of the specimen and the vascular traces, we predicted the order of rootlet development during early ontogeny (Fig. 6D, E). The first rootlet to emerge is the outsized tap root. We could then calculate divergence angles from the taproot to the successive five rootlets. The second rootlet diverges from the rhizomorph at 90° from the first and then the third rootlet emerges between the tap root and this second rootlet. Then, a fourth rootlet emerges at nearly a 180° angle from rootlet 2, and a fifth rootlet emerges at a 74° angle nearly simultaneously between rootlets 2 and 4. Lastly, there is a sixth rootlet that emerges between the fourth rootlet and the tap root at 141° from rootlet 5 (Fig. 6D; Table 2). Based on this analysis, we can reach two important conclusions about rootlet initiation in *Oxroadia*. First, the divergence angle between successive rootlets was highly variable, spanning 58–175°. This means that the pattern cannot be clearly described as a single helix progressing in a clockwise or anticlockwise direction as suggested by Long (1971). Second, the best description of this pattern therefore is not based on divergence angles but rather on the relative position of each rootlet from the preceding ones. We illustrate this with a schematic in Fig. 6E. Development begins with the relatively large tap root (Fig. 6, TR/1) followed by rootlet 2. Following these two rootlets, a clear pattern appears where new rootlets are considered in pairs. Each new pair of rootlets occupies a position that bisects the preceding two. Rootlets 3 and 4 bisect the first two rootlets and then rootlets 5 and 6 bisect 3 and 4 (Fig. 6E). This shows that rootlet development is heavily influenced by the positions of the previous ones.

**Table 2.** The angle of rootlet emergence on the unbranching rhizomorph of the juvenile *Oxroadia* sp. Six rootlets emerge from the rhizomorph where the tap root (TR) is the first-formed rootlet.

Rootlet	TR/1	2	3	4	5	6
Angle of emergence from the previous rootlet (degrees)	N/A	93	58	179	74	141

### Intercalated rootlet development

Based on our investigation, we identify that rhizotaxy in all isoetaleans is best described as a triangular lattice. When rootlets are generated at high densities, as in *Isoetes* and *Stigmaria*, the local pattern is hexagonal or pentagonal. In *Oxroadia*, where rootlet density is low, rootlets may appear in helices or, when enough rootlets are present, a pattern emerges where central rootlets sit in between rootlets above and below (Fig. 6). Based on the juvenile specimen of *Oxroadia* sp., it is clear that there are no set divergence angles between successive rootlets; instead, the position is best described relative to those of the rootlets preceding them (Fig. 6).

This mode of rootlet development is very similar to that described in extant *Isoetes*. A direct comparison can be drawn between the development of the first six rootlets of *I. lacustris*, *I. engelmannii* and *I. tuckermanii* (Hofmeister, 1862; Paolillo, 1963; Karrfalt and Eggert, 1978) and those of the juvenile *Oxroadia* sp. specimen. In all cases there is an initial primary and a secondary rootlet that, together, dictate the positions of the next four rootlets. This pattern, where the preceding rootlets play a key role in dictating the future position of rootlets, also occurs in mature plants of *Isoetes* and was termed intercalation by Paolillo (1982). Although intercalation has been described in mature plants, to our knowledge, it has not been illustrated through a time series of a single plant.

**Fig. 7.**
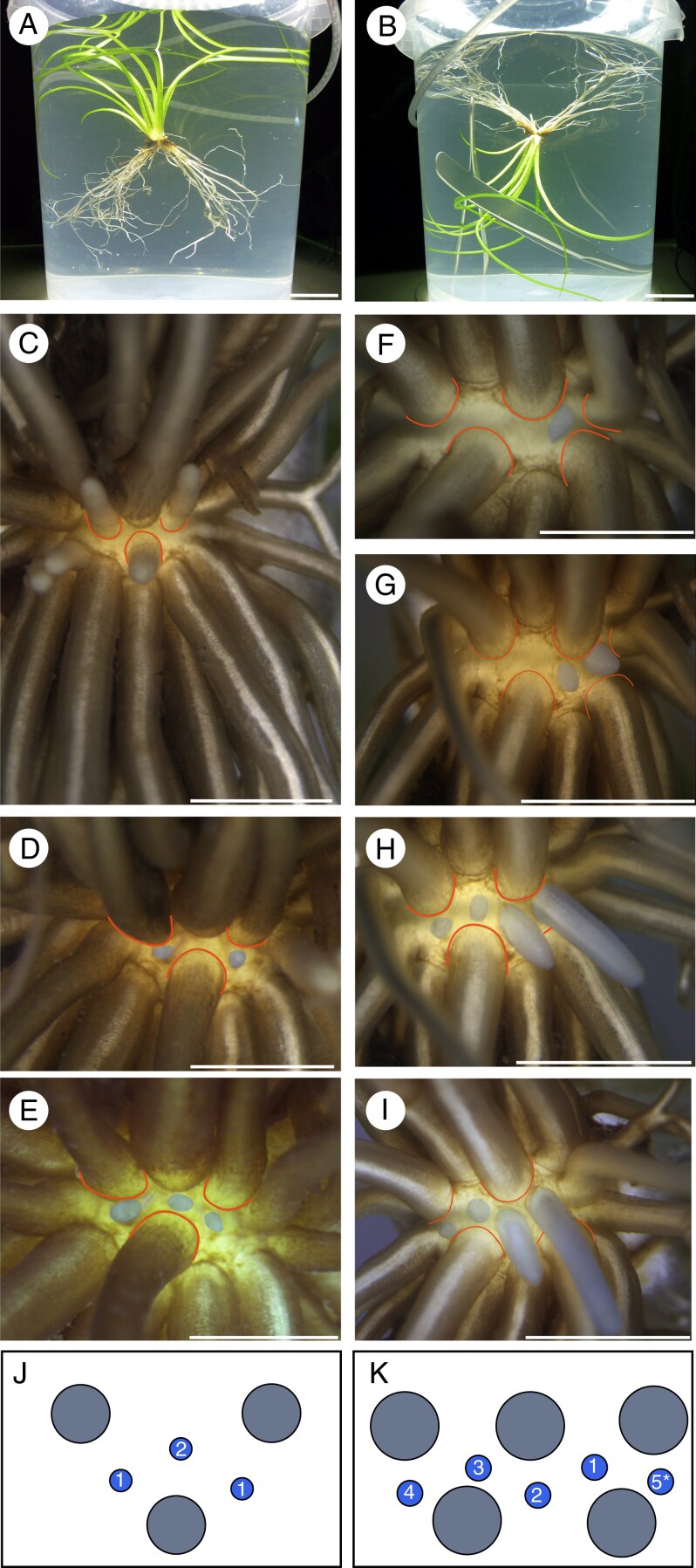
Time-course imaging was used to visualize intercalated rootlet development in two individuals of *Isoetes echinospora*. (A–B) The bilobed, cormose rhizomorph of *Isoetes echinospora* imaged while still submerged. (G–I) The growth series for Plant 1, Day 1 (E), Day 7 (D) and Day 10 (E). In total, three rootlets emerged, the first two simultaneously and the third between the first two. (F–I) The growth series for Plant 2, Day 1 (F), Day 3 (G), Day 5 (H) and Day 6 (I). In total, five rootlets emerged during this time frame; the fifth rootlet (asterisked) is hidden by the angle of the rootlets. The rootlets appear sequentially right to left except for rootlet 5, which appears to the right of rootlet 1. In both cases, rootlets emerge as a new series and intercalate into the existing orthostichies of the mature rootlets. (J–K) Interpretive drawings of rootlet development; mature rootlets are represented by grey circles. The new series of rootlets is shown in blue with the numbers denoting their relative timing of emergence. Scale bars: (A, B) = 2 cm; (C, H, I) = 1.2 mm; (D–G) = 2.4 mm.

**Fig. 8.**
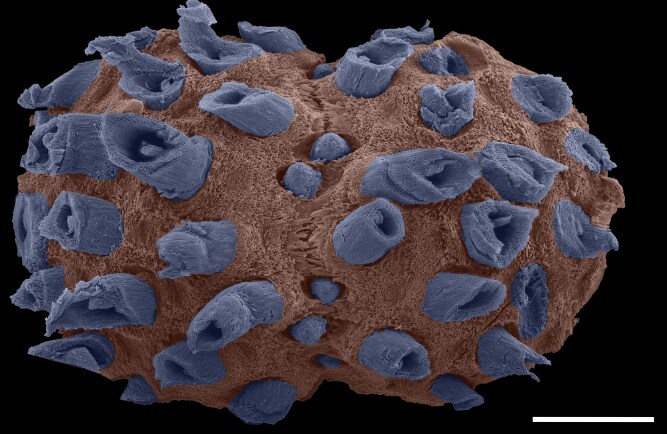
A false-coloured SEM image of *Isoetes echinospora* showing rhizotaxy and developing rootlets on the corm. Rootlets were cut close to their base to help better visualize their relative positions. The rhizomorph is highlighted in red and the rootlets are highlighted in blue. Six younger rootlets are emerging near the linear meristem. Scale bar: 1.5 mm.

To visualize intercalated development live, we imaged rootlet initiation in two *I. echinospora* plants through a 10-d period (Fig. 7A, B). This approach allowed us to visualize the development of a new series of rootlets emerging from the central furrow into the spaces between two existing rootlets and revealed some variation. For example, in Fig. 7F–I the rootlets emerged progressively from left to right, whereas in Fig. 7C–E two rootlets emerged simultaneously with a third developing in between. However, an important unifying feature of all these examples was that rootlets emerged into spaces between older rootlets, therefore intercalating into an existing rhizotaxy. These new images of development through time help interpret the variability in rootlet maturity (Fig. 8), as well as elucidating previous reports of rootlet emergence in the literature (Paolillo, 1963; Karrfalt and Eggert, 1978).

Combining our rhizotaxy analysis across the Isoetales with direct observation of development in *Isoetes* shows that the regular rhizotaxy in the group can be best described based on morphology as a triangular lattice, and that, developmentally, this lattice is the result of intercalation. Rootlets develop as a new series of a similar age; in mature *Isoetes* these series may consist of five or six new rootlets, whereas in juvenile *Isoetes* (Paolillo, 1963) and *Oxroadia*, each new series consists of only one or two rootlets. Although the precise order of emergence of the rootlets in a series can vary (cf. Figs 7 and 8), the rootlets will always form between successive more mature rootlets, thereby intercalating into the existing rhizotaxy. Thus, an explanation becomes apparent for the ‘hexagonal’ organization of rootlets observed by previous authors in both fossil (Charlton and Watson, 1981; Rothwell and Erwin, 1985) and living isoetaleans (Paolillo, 1982).

## DISCUSSION

Our quantitative comparative assessment of rhizomorphic rooting systems identified three areas of interest highlighted by our new findings; here, we consider the technical, palaeoecological and evolutionary implications.

### Potentially widespread applications for a rhizotaxy quantification pipeline

The availability of increasingly sophisticated methods to capture 3D data from both living and fossil species makes it easier than ever before to quantitatively assess the patterning of lateral structures on plant axes. Our data analysis pipeline presented here demonstrates how 3D imaging approaches at different scales, combining SEM imaging, photography and 3D reconstructions of peels, can facilitate comparisons between living and fossil plants. The application of this technique is used here to investigate rhizotaxy, but this same pipeline could be used to quantitatively assess phyllotaxy and the arrangement of reproductive structures, as well as larger-scale branching patterns (cladotaxy). This approach holds particular promise for evaluating other Palaeozoic lycopsids whose phyllotaxy has not been extensively quantified. Isoetalean lycopsid phyllotaxy has been often been simplified to ‘lepidodendroid’ or ‘sigillaroid’ (Meyen, 1976; Wang *et al*., 2005), but this precludes other members of Isoetales such as *Pleuromeia* which have been determined to have a Fibonacci spiral phyllotaxy (Church, 1920; Looy *et al*., 2021) or highly variable with non-Fibonacci spirals present (Dickson, 1873; Schoute, 1938). There are also differences in perspective between characterizing phyllotaxy in permineralized versus adpressed specimens (Watson *et al*., 1987; Spiekermann *et al.,* 2018). Using a geometric approach can unify these descriptions.

Re-evaluating phyllotaxy with a new quantification method is important as it has been used as a taxonomically diagnostic character (Thomas and Meyen, 1984; Wang *et al*., 2003). Expanding our understanding of isoetalean phyllotaxy may improve the understanding of its development, especially in the Devonian taxa. Carboniferous arboreous lycopsids show that leaf cushion shape can alter substantially during ontogeny, notably across transitions from stem to branch and larger branches to smaller branches (e.g. Opluštil, 2010), so we cannot rule out the possibility of parallel shifts in phyllotactic patterning.

Even before the Carboniferous, lycopsids were thriving in the Devonian, developing both herbaceous and arborescent forms (Thomas, 1992; Pigg, 2001; Xu *et al*., 2012). Quantitative assessment of phyllotaxy in the Devonian has been investigated in the two extinct lineages of Devonian lycopsids, Drepanophycales and Protolepidodendrales (Benca *et al*., 2014; Turner *et al*., 2023). Nonetheless, comparing the phyllotaxy of additional taxa using this method should further our understanding of how phyllotaxy evolved across the lycopsid clade during this critical period of lycopsid evolution. The application in palaeobotany of our new approach and similar pipelines offers opportunities to develop new quantitative characters for use in developmental, taxonomic and ecological interpretation.

### Reconsideration of the palaeoecology of *Oxroadia*

Our re-investigation of the rooting system of *Oxroadia* suggests that it is unique among other members of the Isoetales. The rhizomorph of *Oxroadia* is axial, and its rooting system is magnitudes smaller in areal extent than its aerial axes (Fig. 2B); its vasculature is highly branched with clear evidence of anisotomous branching, emitting rootlets of variable diameter. Although the form of a rhizomorph can offer a poor proxy for growth environment, as typified by the ability of *Isoetes* to generate identical rhizomorphs and rhizotaxy in fully aquatic, emergent and fully terrestrial forms (Scott and Hill, 1900; West and Takeda, 1915; Karrfalt and Eggert, 1978), we nonetheless suspect that *Oxroadia*’s unique rooting system was an adaptation to its unstable environment.

The palaeoenvironment of Scotland changed drastically between the late Tournaisian and the late Visean as semi-arid, volcanically influenced environments gave way to wetter, periodically peat-forming environments. *Oxroadia* was a significant component of the floras inhabiting these changing and dynamic ecosystems. In a borehole through the Tournaisian Ballagan Formation of southern Scotland, repeated concentrations of *O. conferta* megaspores showed it to be one of the dominant plant species whereas *O. gracilis* spores were conspicuously absent (Reeves, 2019). Bateman (1992) tentatively assigned a forking axis in a calcareous nodule to *O. conferta* at the diverse early Tournaisian, French Montagne Noire site. At the late Tournaisian Oxroad Bay site studied here, *O. gracilis* and *O. conferta* occur allochthonously in mutually exclusive beds (Bateman and Scott, 1990), indicating that they were deposited at slightly different times and characterized contrasting communities. At this site, *O. conferta* appears to be one component of a diverse flora whereas *O. gracilis* occurred in large monospecific communities (Bateman and Scott, 1990; Bateman, 1992). In contrast with Oxroad Bay, the contemporaneous assemblage at nearby Castleton Bay has *O. gracilis* co-occurring with other lycopsids, including the anatomically similar ‘*Lepidodendron*’ *calamopsoides* (Scott and Galtier, 1988). Body fossils of *O. gracilis* at another Scottish fossil locality, Kingswood, also show that it co-occurs with gymnosperms, living on the shores of a volcanic crater lake (Scott, 2024), and the peels containing the *Oxroadia* sp., re-investigated here, contain multiple species of primitive ferns and pteridosperms. Therefore, *Oxroadia* appears to represent a major component of a wide range of Mississippian communities in Britain and potentially across Europe, occurring alongside a diverse range of other plant groups and populating a considerable spectrum of palaeoenvironmental settings.

Most of the sites where *Oxroadia* has been found, notably those along the Whiteadder Water in SE Scotland and Montagne Noire in south-central France, were not directly influenced by volcanism. Any hypothesis regarding the apparent ecological success of *Oxroadia* species and their unique rhizomorph should take into account the disparate environments in which these plants have been found. Despite having a comparatively small rooting system for its body size, the development of comparatively large-diameter rootlets probably enabled it to establish in unstable substrates such as the volcanigenic sediments at the Oxroad Bay and Kingswood localities, as well as waterlogged, peat-forming environments such as at the Pettycur locality. *Oxroadia*’s comparatively small underground axes also indicate that it probably invested significantly more in its aerial axes than its rooting system. The emergence of the initial ‘tap root’ and subsequent relatively large rootlets near the proximal parts of the rhizomorph probably served an additional, important role in anchoring the plant. In *Stigmaria*, rootlets on lateral branches grew both upwards and downwards from the rhizomorph, but it appears that, like *Isoetes* and other cormose isoetaleans, the rootlets of *Oxroadia* solely grew downwards. We agree with Bateman (1992) that *Oxroadia* was adapted to unstable terrains in environments that varied between seasonally dry and wet conditions. The large and presumably long-lived ‘tap root’ probably was key for becoming established in unstable environments; the growth of rhizomorph and rootlets downwards may well have been adaptations for water uptake, with the decrease in rootlet size on more distal segments of the rhizomorph indicating a shift in functional priority from anchorage to water uptake.

### Conserved rootlet initiation and rhizotaxy

Our analysis defines a basic rhizotaxy that can be applied to all isoetaleans despite their distinct and diverse rhizomorphs. Morphologically, rootlet arrangement can successfully be described via a triangular lattice. Within the lattice structure we can identify parastichies, series and orthostichies (Fig. 3). Moreover, this regular pattern is underpinned by specific development where new rootlets intercalate into the existing lattice, forming on intermediate orthostichies between preceding rootlets. These observations suggest that rootlet development in *Isoetes* and *Oxroadia* is not determined by a specific divergence angle from the proceeding rootlet but is instead determined relative to the preceding series of rootlets. Our time-series images demonstrate that this mode of development characterizes *I. echinospora*. New rootlets appear in series that are developmentally almost synchronous in age (Paolillo, 1963, 1982; Karrfalt and Eggert, 1978), analogous to a new series of leaf primordia initiated in a whorl. However, the rootlets do not emerge synchronously, as rootlet development is endogenous within the corm (Yi and Kato, 2001); rootlets proceed with differential vigour and so emerge at different times (Figs 7 and 8), intercalating into the existing rhizotaxy. In a mature *I. echinospora* plant, a new series can include at least six new rootlets (Fig. 8), whereas, at the early stages of development, a series may only be composed of one or two rootlets (Paolillo, 1963, 1982; Karrfalt and Eggert, 1978). This low series number appears most consistent with our results from *Oxroadia*, where rootlets reliably occur at low densities in both the juvenile and mature specimens. By contrast, we do not currently have any direct evidence that would allow us to conclude about the exact mode of rootlet development in *Stigmaria*. If *Stigmaria* rootlet initiation was similar to *Isoetes* and *Oxroadia*, the very high density of rootlets in *Stigmaria* probably resulted from the more-or-less simultaneous initiation of a large number of rootlets. Charlton and Watson (1981) predicted that a rhizomorph apex might result in the initiation of 14–17 primordia simultaneously but also stated that it was not easy to imagine how this could occur. The subsequent description of well-preserved apices of stigmarian axes (Rothwell, 1984; Rothwell and Pryor, 1991) that featured a large ring-shaped meristem suggest that the size of the meristem was probably easily capable of initiating series involving large numbers of primordia (DiMichele *et al*., 2022).

This mode of development is most similar to whorled or non-Fibonacci phyllotaxy such as quasi-symmetrical arrangements on shoots reported for both fossil (Alvin, 1965; Meyer-Berthaud, 1984; Bateman, 1992; Wang *et al*., 2005; Spiekermann *et al*., 2018; Prestianni *et al.*, 2022) and extant lycopsids (Charlton and Watson, 1981; Vindt-Balguerie, 1991; Douady and Golé, 2016; Gola and Banasiak, 2016; Golé *et al*., 2016; Turner *et al.*, 2023; Walch, 2025). Our understanding of the development of quasi-symmetrical (Douady and Golé, 2016; Golé *et al*., 2016), also referred to as *n*:(*n* + 1) (Charlton and Watson, 1981; Turner *et al*., 2023), phyllotaxy would greatly benefit from future investigations in the shoots of extant lycopsids. Specifically, these investigations will shed light on the possible modes of development that can produce *n*:(*n* + 1) phyllotaxy in shoots of lycopsids and therefore may allow us to infer the mode of development leading to the *n*:(*n* + 1) rhizotaxy observed in *Stigmaria* (Charlton and Watson, 1981). Based on our data here it will be valuable to establish if new leaves initiating in *n*:(*n* + 1) phyllotaxy follow an ontogenic spiral with regular divergence angles between successive primordia or if leaves initiate in a series with variable divergence angles. These investigations will help to better constrain the developmental mechanism that led to the rhizotaxy observed in isoetaleans. This will be aided by the discovery and description of further *Oxroadia* specimens, and apical portions of *Stigmaria*. The paucity of *Oxroadia* rhizomorph fossils made definitive rhizotaxy characterization difficult. The order in which rootlets emerged from the juvenile specimen’s rhizomorph suggests that they formed in a series, but the observations of simple helical arrangements on some branches described here may indicate that, in some cases, rootlets were initiated in a helical rhizotaxy. Even with more fossil specimens, it may still be difficult to interpret *Oxroadia*’s rhizotaxy simply due to the highly branched nature of its rhizomorph, which limits the number of rootlets per segment. However, our study demonstrates a method that will aid further investigations of both phyllotaxy and rhizotaxy when new fossils are discovered.

Despite the increasingly well-documented contrasts in morphology among isoetalean rhizomorphs, it appears that their rhizotaxy may have a common developmental mechanism, and that this mechanism bears close similarities to the development of microphyllous leaves in some living and extinct lycopsids, especially living members of the Lycopodiaceae (Charlton and Watson, 1981; Gola, 1996; Douady and Golé, 2016; Golé *et al*., 2016; Turner *et al*., 2023; Walch, 2025). Admittedly, there remains the key difference that leaves develop at the shoot apex whereas rootlets develop endogenously from the rhizomorph.

### Ontogeny of *Oxroadia* and implication for the ‘modified shoot’ hypothesis

Our analysis of rhizotaxy across the isoetaleans provides a welcome opportunity to revisit a major hypothesis in the literature for the origin of the rhizomorph, termed the ‘modified shoot’ hypothesis. Stated baldly, this hypothesis suggests that the evolutionary origin of the rhizomorph was the result of direct evolutionary modification of a leafy shoot early in development. Specifically, after germination, a unipolar embryo developed and subsequently underwent an early dichotomy, giving rise to shoot and rhizomorph axes respectively. The direct evidence supporting this interpretation comes from observations on a young sporophyte developing largely within the wall of a megaspore of the phylogenetically highly derived tree-clubmoss *Lepidophloios* (Stubblefield and Rothwell, 1981; Rothwell and Pryor, 1991). The expanded hypothesis that the rhizomorphs of all isoetaleans represent shoots is built on the inference that the same organization described in *Lepidophloios* was ancestral in the clade. This interpretation is at odds with evidence from the embryogeny of extant *Isoetes* (Bower, 1935), or predicted for the fossil *Bothrodendrostrobus* (Stubblefield and Rothwell, 1981). The embryos of both appear far more similar to those of other extant lycopsids with distinct foot, embryonic first root and cotyledon than to the embryo of *Lepidophloios*. Studies of development in extant *Isoetes* demonstrate that the rhizomorph meristem appears after the development of the first few rootlets (Yi and Kato, 2001) with no evidence of a primary dichotomy of the shoot. The ‘modified shoot’ hypothesis therefore predicts that this initial dichotomy of the embryo was lost in *Isoetes* during the evolution of a cormose growth habit. However, an alternative interpretation is that the unipolar embryo preserved in *Lepidophloios* is in fact a highly derived condition associated with the evolution of extreme heterospory in *Lepidophloios*, analogous to changes in embryogeny predicted to accompany the evolution of the seed in euphyllophytes. *Oxroadia*’s early diverging position within the isoetaleans makes it an ideal test case to investigate embryo structure and early sporophyte ontogeny.

Bateman (1992) described an embryo from *Oxroadia* that demonstrated no evidence for an initial unipolar embryo with initial dichotomy. Instead, he documented evidence for a continuous vascular strand, one end representing the shoot and the other end probably representing an embryonic root (the tap root). The embryogeny was more similar to that of extant *Isoetes* and demonstrates that this organization could give rise to radially symmetric rhizormorphs, therefore not requiring an initial embryonic dichotomy (Bateman, 1992). Here we investigated the juvenile *Oxroadia* sp. specimen in search of additional evidence of a primary dichotomy of the shoot early in development. We found no evidence for a horizontal vascular strand where the tap root emerges at the transition between the rhizomorph’s siphonostele and the stem’s haplostele (Supplementary Data Fig. S3). This critical but easily overlooked structure was tentatively identified by Rothwell and Erwin (1985) within the wood of a mature specimen of the small-bodied Kazimovian–Gzhelian lycopsid *Paurodendron*. If present in *Oxroadia*, we expected this feature to be much more prominent in a juvenile specimen, or to have been identified in our re-investigation of the mature specimens of *O. gracilis* or *O. conferta.* These lines of evidence provide no support for the hypothesis that *Oxroadia* developed from an embryo with an initial embryonic dichotomy.

Our analysis therefore finds no support for the literal interpretation that the rhizomorph is a ‘shoot modified for rooting’ (Stubblefield and Rothwell, 1981; Rothwell and Pryor, 1991). We instead favour the suggestion by Bateman *et al*. (1992) that the rhizomorph be interpreted as a ‘shootlike developmental system but prefer to regard it as a unique organ’ – a view supported by Gensel and Berry (2001) and Hetherington *et al.* (2020). Admittedly, our analysis of rhizotaxy adds further evidence to the similarities between the development of the rhizomorph and shoots in lycopsids. However, we interpret this to represent the co-option of an existing developmental programme in a novel organ, rather than a literal evolutionary transition of a leaf-bearing shoot into rootlet-bearing rhizomorph. We hope this interpretation stimulates the discovery of further direct evidence for early stages of development in the Isoetales and re-centres the debate on the evidence for lycopsid rooting system evolution outside of the rhizomorphic clade.

### Re-centring the debate about the origin of the rhizomorph

The debate concerning the homology of the rhizomorph has overshadowed how little is still known about the evolution of rooting systems along the entire backbone of the lycopsid phylogeny. The fossil record of the rhizomorphic lycopsids, revealing a diversity of rhizomorph types spanning unbranched, lobed, cormose forms and the first stigmarian rooting systems, extends from the Middle–Late Devonian onwards (Cressler and Pfefferkorn, 2005; Prestianni and Gess, 2014; Berry and Marshall, 2015; Xu and Wang, 2015; Wang *et al*., 2019; Stein *et al.*, 2021). This is only 50 million years later than the earliest complex rooting systems are preserved in any lycopsids – specifically in the Early Devonian Drepanophycales (Gensel *et al*., 2001; Xu *et al*., 2013; Matsunaga and Tomescu, 2016, 2017; Hetherington and Dolan, 2018; Hetherington *et al*., 2021). Unifying both these distinct groups are rooting systems composed of two parts – small dichotomously branching roots (rootlets of the isoetaleans and the roots or transitional rooting axes of the drepanophycaleans) that develop from root-bearing structures (rhizomorphs of the isoetaleans, and root-bearing axes of the drepanophycaleans). However, separating these distinct groups in a comparatively short time period spans the divergence of the extant clades of the Lycopodiales, Selaginellales and the extinct Protolepidodendrales. The steps in rooting system evolution connecting the drepanophycalean rooting systems with those of the isoetaleans remains unclear and we currently lack good predictions for the rooting systems in the common ancestors of either crown lycopsids or ligulate lycopsids (Matsunaga *et al*., 2017; Fujinami *et al*., 2021; Hetherington *et al*., 2021; Ito *et al*., 2022). Many rooting systems in the extant lycopsids develop a two-part rooting system, separated into roots and root-bearing structures (Hetherington and Dolan, 2017; Hetherington *et al*., 2021). A key goal for the future will be to predict if two-part rooting systems were present in the common ancestors of crown lycopsids and ligulate lycopsids, and whether distinct root-bearing structures are present (Fujinami *et al*., 2021) or have been lost from many members of the Lycopodiales. Making predictions about the rooting systems in the common ancestor of the ligulate lycopsids will be key to establishing whether the characteristics that distinguish the rhizomorph were acquired sequentially before the divergence of the Isoetales.

The rhizophore of *Selaginella* has already been the crux of many debates about rooting system evolution (Imaichi and Kato, 1991; Lu and Jernstedt, 1996; Kawai *et al*., 2010; Matsunaga *et al*., 2017; Hetherington and Dolan, 2017; Mello *et al*., 2019). However, it remains unclear if the rhizophore was ancestral within the Selaginellales (Bateman, 1996*a*, *b*; Korall and Kenrick, 2002). Instead, the common ancestor of living Selaginellales may have developed a rooting system closer to that of *Selaginella selaginoides*, sister to all other members of the Selaginellales (Korall and Kenrick, 2002; PPG I, 2016; Zhou and Zhang, 2023). In overall habit, *S. selaginoides* possesses a rooting system that is small in comparison with its far more extensive shoot system, resembling *Oxroadia* in this feature. Moreover, roots of *S. selaginoides* develop from a unique root-bearing structure termed the ‘basal swelling’ (Karrfalt, 1981). Although the basal swelling differs significantly from the rhizomorph of the isoetaleans, it nonetheless shares some key characters such as developing roots in a set sequence previously recognized as an intercalated pattern (Karrfalt, 1981). This example emphasizes the need for further investigation of rooting systems in a diverse range of lycopsids. We highlight the Protolepidodendrales as a particularly important group for re-investigation in the future, given their potential for predicting the rooting system present in the common ancestor of the ligulate lycopsids. Our pipeline proposed here for the quantitative assessment of rhizotaxy will provide additional characters to investigate the evolution of rooting systems in the lycopsids.

## Supplementary Material

mcaf277_Supplementary_Data

## Data Availability

Data for this work can be found in the paper as well as on the Edinburgh University DataShare (DOI: 10.7488/ds/7972). Fossil specimens described in this study are deposited in three collections: National Museums Scotland; Hancock Museum, Newcastle; University of Birmingham.

## SUPPLEMENTARY DATA

Supplementary data are available at *Annals of Botany* online and consist of the following. Fig. S1. Delaunay triangulation applied to randomly selected sections of the rhizotaxy plots generated from 3D photogrammetry models of three additional *Stigmaria* specimens. (A–C) *Stigmaria* specimens NMS G.1988.15.1, G.2022.11.49 and G.1983.34.2 with blue boxes showing the areas that had Delaunay triangulation applied to them. (D–F) Rhizotaxy plots for A–C with black boxes representing the regions that were enlarged and had Delaunay triangulation applied. The triangulation plots show consistent rhizotaxy even where there is local preservational damage as on the highlighted region of D. Scale bars: 6 cm. Fig. S2. Quantification of the triangular lattice. Mean distribution of angle measurements of triangles within a lattice falls near 60°, demonstrating that the majority of these triangles are equilateral. (A, C, E, G) The full rhizotaxy plots of *Stigmaria* 1–4, respectively, imaged in Fig. S1 with Delaunay triangulation applied. (B, D, F, H) The distributions of each angle of the triangles generated. The number next to the distribution curves is the mean, all of which fall within 10 points of 60 and most of which are within 5 points. This includes the natural distortions such as the more severe angles at the edges and the areas of preservational damage. Fig. S3. Vascular detail of the transition between the stem base and the rhizomorph is well preserved in the juvenile *Oxroadia* specimen, and there is no evidence of a horizontally oriented embryonic strand of xylem consistent with development from a unipolar embryo. (A–L) The series of acetate peels captures the entire transition between stem and rhizomorph. Several members of the series were not included in the final figure in order to better show the change in vascular architecture across this transition point. The first rootlet to emerge is the large tap root, and the second rootlet emerges to the left of the tap root. For A–L the peels are respectively AGL 7402, 7401, 7398-96, 7394-90, 7388 and 7386. (M–P) Higher magnification images of peels AGL 7398, 7394, 7391 and 7386, respectively, taken with a Leica EZ4 W microscope. Scale bars: (A–P) = 4 mm.

## AI ASSISTANCE

The authors declare that AI was not used for preparation, translation or writing of the manuscript.
