# Supplementary material for: Comparative rhizotaxy of fossil and living isoetalean rhizomorphs reveals development through rootlet intercalation within a triangular lattice
Source: Ann Bot. 2025 Oct 31;137(6):1709–25. doi: 10.1093/aob/mcaf277 (PMC13274994; doi:10.1093/aob/mcaf277)
Supplement: mcaf277_Supplementary_Data [file mcaf277_supplementary_data.docx]

**Supplemental Data**

**Supplementary Fig. 1-3**


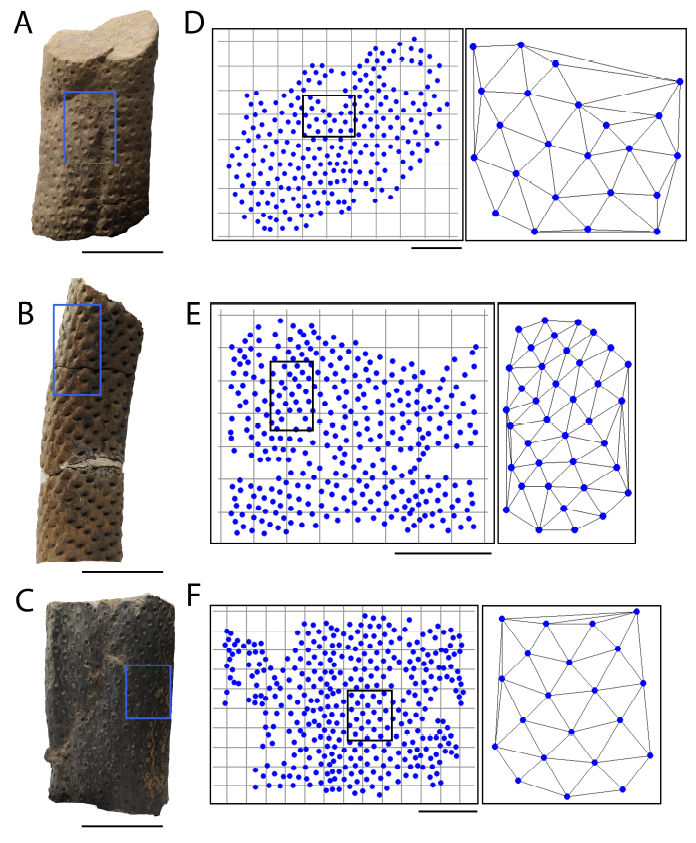
Supplementary Fig. 1. Delaunay triangulation applied to randomly-selected sections of the rhizotaxy plots generated from 3D photogrammetry models of three additional *Stigmaria* specimens. (A–C) *Stigmaria* specimens NMS G.1988.15.1, G.2022.11.49 and G.1983.34.2 with blue boxes showing the areas that had Delaunay triangulation applied to them. (D–F) Rhizotaxy plots for (A–C)with black boxes representing the regions that were enlarged and had Delaunay triangulation applied. The triangulation plots show consistent rhizotaxy even where there is local preservational damage like on the highlighted region of (D). Scale bars: 6 cm.


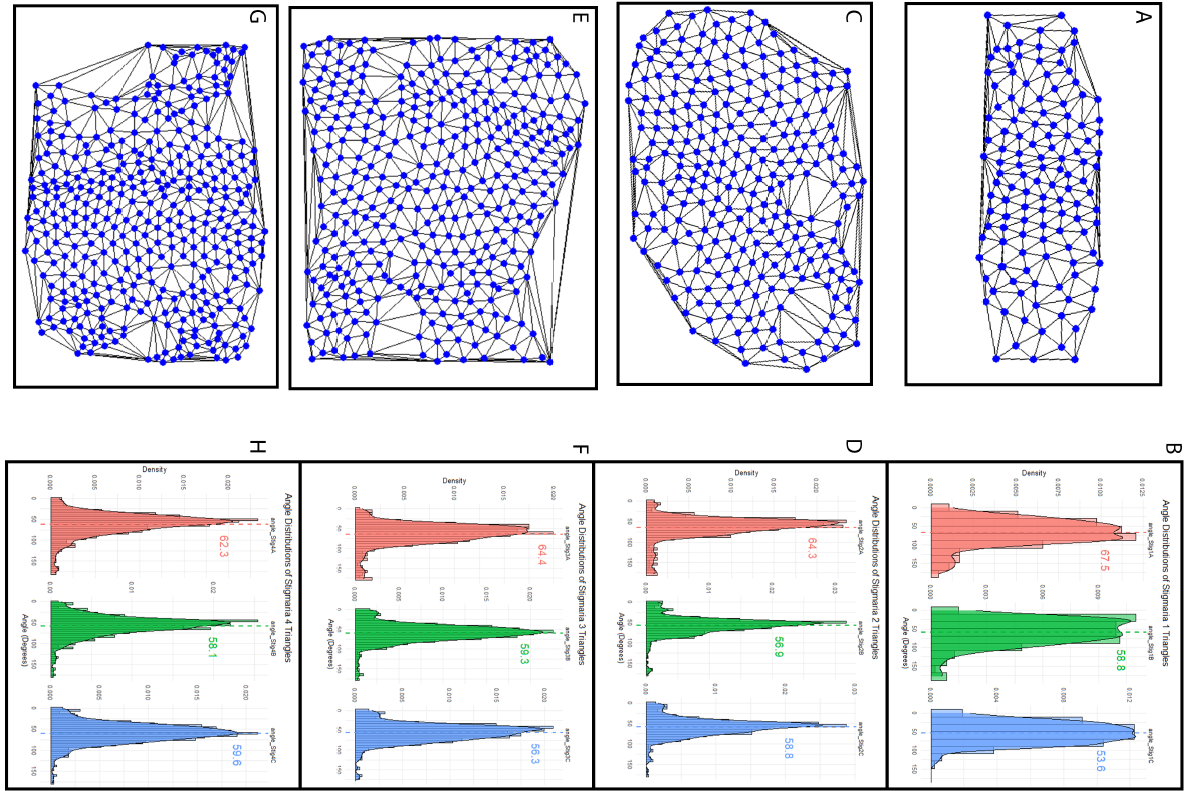


Supplementary Fig. 2. Quantification of the triangular lattice. Mean distribution of angle measurements of triangles within lattice falls near 60 degrees, demonstrating that the majority of these triangles are equilateral. (A, C, E, G) The full rhizotaxy plots of *Stigmaria* 1-4, respectively, imaged in Supplementary Fig. 1 with Delaunay triangulation applied. (B, D, F, H) The distributions of each angle of the triangles generated. The number next to the distribution curves is the mean, all of which fall within 10 points of 60 and most of which are within 5 points. This includes the natural distortions such as the more severe angles at the edges and the areas of preservational damage.


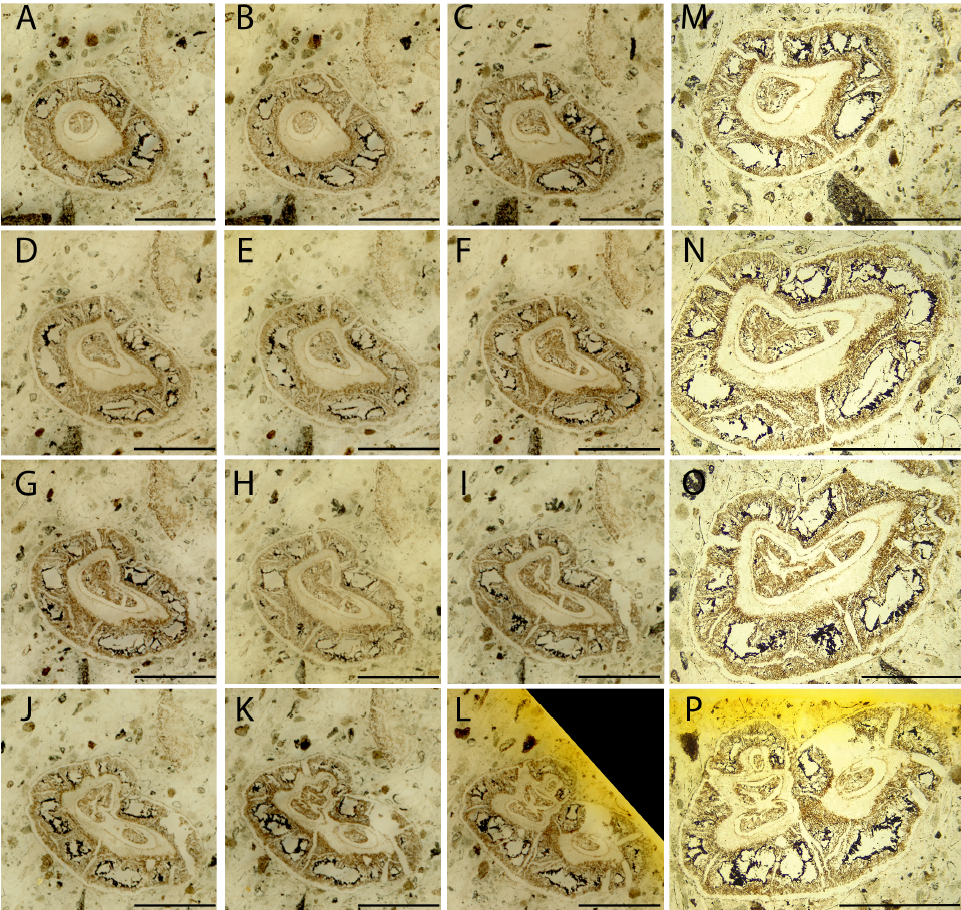
Supplementary Fig. 3. The vascular detail of the transition between the stem base and the rhizomorph is well-preserved in the juvenile *Oxroadia* specimen, and there is no evidence of a horizontally oriented embryonic strand of xylem consistent with development from a unipolar embryo. (A–L) The series of acetate peels captures the entire transition between stem and rhizomorph. Several members of the series were not included in the final figure in order to better show the change in vascular architecture across this transition point. The first rootlet to emerge is the large tap root, and the second rootlet emerges to the left of the tap root. For (A–L) the peels are respectively AGL 7402, 7401, 7398-96, 7394-90, 7388, 7386. (M–P) Higher magnification images of peels AGL 7398, 7394, 7391 and 7386 respectively, taken with a Leica EZ4 W microscope. Scale bars: (A–P) = 4 mm.
